# Global big data laboratory experiment, integrated with kernel-based algorithm with an improved nonlinear ensemble for compressive strength modeling

**DOI:** 10.1038/s41598-024-58908-9

**Published:** 2024-12-28

**Authors:** Babatunde Abiodun Salami, Jamilu Usman, Afeez Gbadamosi, Salim Idris Malami, Sani I. Abba

**Affiliations:** 1 Cardiff School of Management, Cardiff Metropolitan University, CF5 2YB Cardiff, Wales, United Kingdom; 2 Interdisciplinary Research Center for Membrane and Water Security, King Fahd University of Petroleum and Minerals, 31261 Dhahran, Saudi Arabia; 3 Department of Petroleum Engineering, College of Petroleum Engineering and Geosciences, King Fahd University of Petroleum and Minerals, 31261 Dhahran, Saudi Arabia; 4 Department of Civil Engineering, Kano University of Science and Technology, Wudil, Kano, Nigeria

**Keywords:** Blended concrete, Support vector regression, Robust linear regression, Machine learning, Ground granulated blast furnace slag, Civil engineering, Environmental sciences, Engineering

## Abstract

With the continuous clamor for a reduction in embodied carbon in cement, rapid solution to climate change, and reduction to resource depletion, studies into substitute binders become crucial. These cementitious binders can potentially lessen our reliance on cement as the only concrete binder while also improving concrete functional properties. Finer particles used in cement microstructure densify the pore structure of concrete and enhance its performance properties. The compressive strength of concrete made from a mixture of ground granulated blast furnace slag (GGBFS), fly ash (FA), and ordinary Portland cement was estimated using kernel regression techniques in this work. The kernel-based method offered was support vector regression (SVR), while robust linear regression (RLR), and multi-linear regression (MLR) were used as regression methods, subsequently, nonlinear average approaches were used to improve the accuracy of the prediction. Eight variables (cement, FA, GGBFS, water, superplasticizer dose [SP], coarse aggregate [CA], fine aggregate [F_ag_], age) were employed as input features in 3323 data samples, and their relative value was assessed using linear correlation analysis. Following analysis, three combinations were employed to train the kernel-based models: I (inputs: cement, water, and age|output: CS), II (inputs: cement, water, FA, SP, and age|output: CS), and III (inputs: cement, water, FA, SP, CA, GGBFS, and F_ag_|output: CS). The third combination gave the best testing performance with all the proposed models where their R^2^ and MSE results after model evaluation for SVR, RLR, and MLR, are [0.984, 0.8776 and 0.8804] and [0.0019, 0.0131 and 0.0128] respectively. The study concludes that SVR with the combination III (SVR-M3) offered the best performance through effectiveness and efficiency in accurately predicting the compressive strength of the blended concrete. The prediction models should be utilized with the input variable ranges used in this work.

## Introduction

Owing to its compositional assemblage of water, cement, and numerously available additives, conventional concrete is the most frequently employed material in structural applications. The water-to-cement ratio, amount of cement used, level of moisture, and kind of additives are the key determinants of strength^1^. For the development of reinforced concrete buildings, one of the most often utilized metrics in structural engineering is still concrete’s compressive strength. When the concrete compressive strength test is employed as a destructive operation on concrete specimens, the concrete’s performance if empirically defined, can be altered by non-linear variables. However, since the generally utilized compressive strength factor is only discovered on the 28th day, this activity requires time, preparation, and financial resources^2^.

New research has been prompted to create new types of binders and prediction models capable of precisely estimating their concrete qualities by the fast development of novel forms of mixed concrete, encouraged by the ever-growing requirements from the building and construction industry^3–6^. Due to its link with other parameters related to mechanical performance and durability of concrete, compressive strength continues to be the most crucial component of concrete. Many studies have investigated the effects of many influential parameters on the compressive strength of concrete, be it traditional concrete, self-compacting concrete (SCC)^7–9^, high performance concrete (HPC)^10,11^, etc. In a SCC study, some researchers^9^ developed a binary concrete where Portland cement was replaced with limestone powder (an additive), then tests were conducted to assess its compressive strength and durability performance. Current approaches for estimating the compressive strength of concrete include a combination of linear and non-linear regression techniques such as artificial neural network (ANN), emotional neural network (ENN), Extreme Learning Machine (ELM), Minimax Probability Machine Regression (MPMR), and others. For example, Abounia-Omran et al.^12^ used four regression tree models (M5P, REPTree, M5-Rules, and decision stump) and two ensemble approaches (additive regression and bagging) to estimate the compressive strength of concrete containing three different materials (fly ash, Haydite lightweight aggregate, and portland limestone cement). The results showed some acceptable prediction performance with coefficient of determination (R^2^) ranging from 0.92 to 0.98.

Behnood and Mohammadi Golafshani^13^ used hybrid artificial neural network (HANN) with multi-objective grey wolves (MOGWO) to predict the compressive strength of concrete with silica fume. With a Pearson correlation coefficient of 0.96, the authors concluded on the robustness of HANN optimized by MOGWO in accurately predicting the compressive strength. In order to evaluate the compressive strength of concrete, fuzzy logic was also applied. The findings were discovered to be accurate with a manageable amount of error^14^.

Moreover, kernel-based regression algorithms have also been reportedly used in estimating different properties of concrete. In a kernel-based regression study, Verma et al. employed three different models (Gaussian process regression (GPR), SVR and relevance vector machine (RVM)) to estimate the 28-day compressive strength of cement with C_3_S (%), SO_3_ (%), alkali (%) and Blaine (cm^2^/g) as inputs. Particle swarm optimization (PSO) and symbiotic organism search (SOS) are two separate metaheuristic optimization methodologies used to obtain SVR hyperparameters. The RVM and GPR hyperparameters are determined through trial and error. The authors concluded that the kernel-based models offer a better prediction tool for evaluating cement strength due to their superior generalization capability and great empirical performance. Evidence of kernel-based models’ reliability and extensive deployment in predicting target parameters abound and have been well reported^15,16^ not only in concrete materials research^17,18^ but also in other areas^19,20^. While previous researchers have examined the efficacy of recent machine learning (ML) techniques utilising diverse optimisation algorithms for predicting the compressive strength of concrete in various applications^21–23^, our study makes use of kernel-based regression and nonlinear ensemble models. Kernel regression models are a type of machine learning approach that can be utilised for making predictions about continuous variables such as the compressive strength of concrete. Nonlinear ensemble models are another type of machine learning technique that can be employed to enhance the precision of other machine learning models by combining them.

To broaden the scope of predictive modeling, our study considered the intricate composition of blended concrete, which is made up of FA, GGBS, OPC, water, and superplasticizer to estimate the 28-day compressive strength. Three kernel-based regression models, support vector regression (SVR), robust linear regression (RLR), and multilinear regression (MLR), were put into use to analyze a large dataset containing more than 3,300 distinct concrete mixture designs. This is a much larger dataset than has been used in previous studies on the compressive strength of concrete. Based on their correlation coefficient values, our creative method involved combining a variety of input factors. The accuracy of our prediction models was also much improved by the use of nonlinear ensemble approaches. This method demonstrated a departure from the custom of depending on a single prediction model. Our comprehensive comparison of various predictive models furnished valuable insights into their relative efficacy. This integrative perspective, which prioritizes precision and optimality, has enriched our comprehension of compressive strength estimation in concrete. Our discoveries constitute a minor improvement in the realm of construction material science and hold little potential to revolutionize the course of future construction material development.

## Proposed methodology

**Figure 1:**
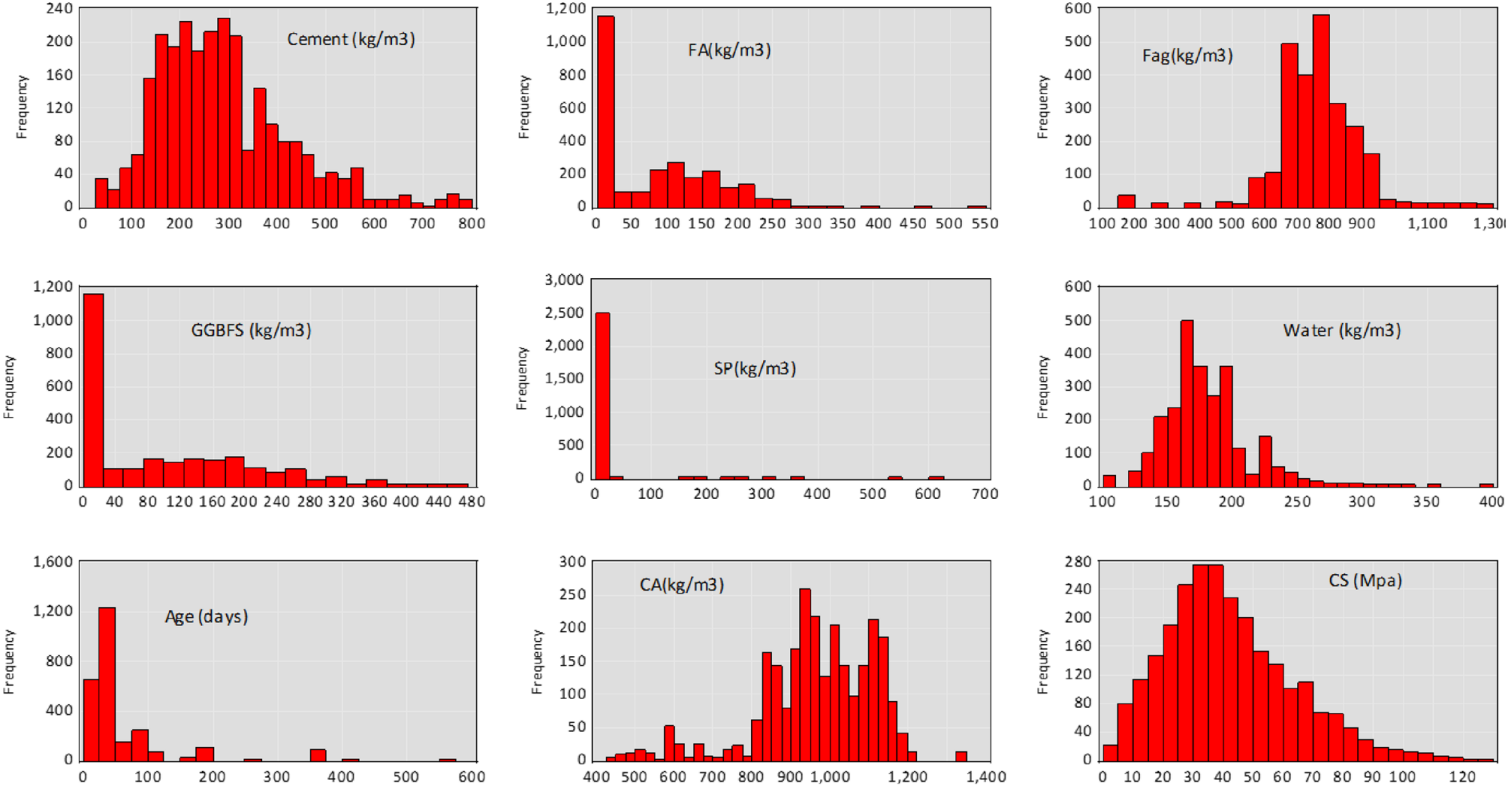
The distribution of the raw data used in this study.

**Figure 2:**
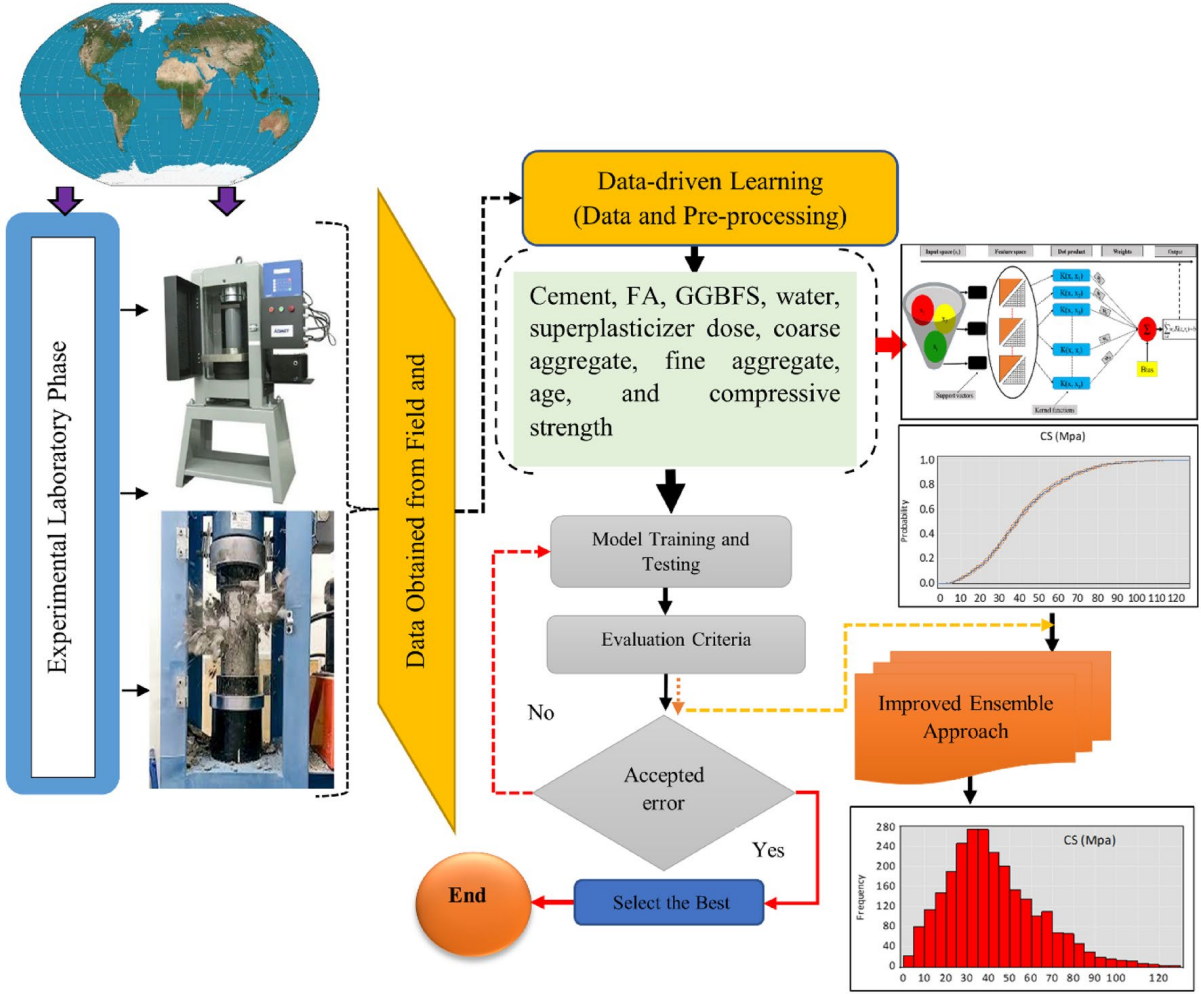
Flowchart for the methods used in the research.

**Figure 3:**
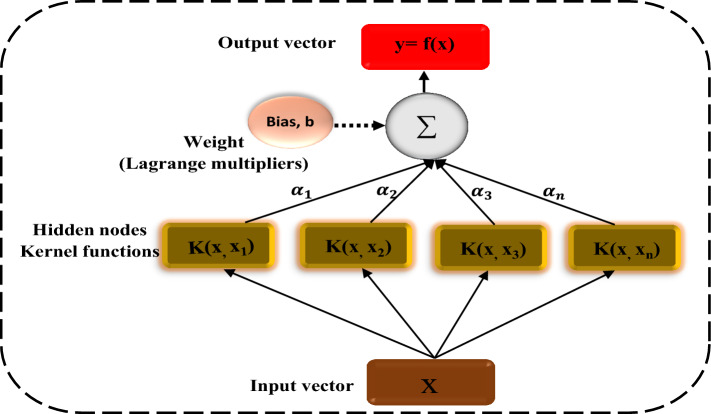
SVR model architecture.

The 3323 datasets for all the studies used in building the database are the long-period compressive strength of concrete made with a blend of FA, GGBFS, and OPC laboratory data taken from a popular repository (1030 data points)^24,25^ and the rest of the data from other works^26,27^. It is crucial to point out that the compressive strength was obtained from cubic specimens in compliance with ASTM C39 guidelines^28^. The laboratory experiment looked at how a mixture of OPC, GGBFS, FA, water, SP, age, and the aggregates affected the produced concrete’s compressive strength. The data that is currently accessible shows that the database sampled a wide variety of different cocktail compositions. We considered the effects of time (age) and material properties (OPC, GGBFS, water, SP, CA, and FA) on the strength output of the blended concrete while constructing our models (Fig. 1). 70% of the experimental dataset was used to train the model, while the remaining 30% of the testing dataset was used for prediction and validation. Figure 3 depicts the flowchart of the suggested models’ training, validation, and testing processes. The sections that follow explain in detail the proposed ML algorithms used in the study. Figure 2 shows the flowchart for the methodology followed in the model development.

### Support vector regression (SVR)

Because of its strong feature of fast adopting statistical learning theory and minimizing the amount of structural risk, a Support Vector Machine (SVM) can complete the distinctive mix of classification, regression, prediction, and pattern recognition of a specific collection of issues^29,30^. Another benefit of SVM over conventional AAN is its potential to boost data network performance. SVMs are categorized into two categories based on their requirements: non-linear support vector regression (N-SVR) and linear support vector regression (L-SVR) (L-SVR). The former is used to tackle direct technical issues, while the latter is used to analyze nonlinear data and construct models^31^

Weighting of input variables is dealt with in the first layer, while weighting of output variables is dealt with in the second layer. For a set of training data $$\left\{ {\left( {x_{i} ,d_{i} } \right)} \right\}$$$$_{i}^{N}$$ Eq. (1) gives the generic SVR function (x_i_, d_i_ and N are the input vector, actual value, total number of data patterns respectively).

1 $$y = f(x) = w\varphi (x_{i} ) + b$$

where φ(x_i_) signifies feature spaces nonlinearly transferred from the input vector x. By giving positive values to the slack parameters ξ and ξ^*^ minimising the important function indicated in Eq. (2), the regression parameters b and w may be obtained:

2 $$\frac{1}{2}\left\| w \right\|^{2} + C\left( {\sum\nolimits_{i}^{N} {(\xi_{i} + \xi_{i}^{*} )} } \right)$$

3 $${\text{Subject}}\,{\text{to:}}\left\{ {\begin{array}{*{20}l} {w_{i} \varphi (x_{i} ) + b_{i} - d_{i} \le \varepsilon + \xi _{i}^{*} } \hfill & {} \hfill \\ {d_{i} - w_{i} \varphi (x_{i} ) + b_{i} \le \varepsilon + \xi _{i}^{*} } \hfill & {{\text{i}} = 1,2, \ldots {\text{N}}} \hfill \\ {\xi _{i} ,\xi _{i}^{*} } \hfill & {} \hfill \\ \end{array} } \right.$$

where $\frac{1}{2}\left\| w \right\|^{2}$ are the weights vector norms and C is the regularized constant Fig. 3 depicts the basic conceptual model structure of SVR. The Lagrange multipliers’ parameters are denoted by α_i_ and α_i_^*^ After solving the optimization problem, the vector w in Eq. (4) may be computed.

4 $$w^{*} = \sum\nolimits_{i = 1}^{N} {(\alpha_{i} - \alpha_{i}^{*} )\varphi (x_{i} )}$$

As a result, Eq. (5) is informed by the overall form of SVR.

5 $$f(x,\alpha_{i} ,\alpha_{i}^{*} ) = \sum\nolimits_{i = 1}^{N} {(\alpha_{i} - \alpha_{i}^{*} )K(x,x_{i} ) + b}$$

The kernel function is k (x_i_, x_j_), and the bias term is b. The most popular kernel function is the Gaussian Radial Basis Function, which is represented as:

6 $$k(x_{1} ,x_{2} ) = \exp ( - \gamma \left\| {x_{1} - x_{2} } \right\|^{2} )$$

where the kernel parameter is γ.

## Robust linear regression (RLR)

The real linear regression (LR) is separated into two types: one for estimating a single predictor with one variable (simple regression) and another for estimating several predictors with one variable (Multiple regression). Multiple regression (multilinear regression) was used in this study’s analysis. Multilinear regression is the most prevalent kind of linear regression^30,32^. Each independent variable value will be allocated to a dependent variable value in this sort of analysis.

### Multilinear regression analysis (MLR)

The least squares approach is often employed for creating a model with a linear connection between two parameters. Multiple linear regression (MLR) is used to establish a link between more than two variables^30,31^. Because of its ease of use, multiple linear regression (MLR) is the most often used strategy in analysis and model building, while simple linear regression (SLR) focuses on constructing a connection between two variables, namely the dependent and independent variables^33^. It’s also seen in a larger perspective below.

7 $${\text{y}}^{{\frac{{1}}{{4}}}} {\text{ = B}}_{{0}} {\text{ + B}}_{{1}} {\text{ X}}_{{1 }} {\text{ + B}}_{{2 }} {\text{X}}_{{2 }} {\text{ + B}}_{{\text{i}}} {\text{ X}}_{{\text{i}}}$$

where X_1_ is the ith predictor’s value, B_0_ is the regression constant, and bi is the ith predictor’s coefficient.

### Nonlinear simple average

The suggested nonlinear simple average (NLSA) or simple average ensembles (SAE) approach involves training and testing the SVR, RLR and MLR models independently, then comparing and testing the average of the SVR, RLR and MLR outputs against the test experimental values^31^. The typical SAE equation is as follows:

8 $${\text{P}}_{{\text{t }}} = \frac{1}{{\text{N}}}{\Sigma }_{{{\text{i}} = 1}}^{{\text{N}}} {\text{ P}}_{{\text{i }}} ({\text{t)}}$$

where N is the total number of learners (here N = 4) and p_i_ is indeed the output of a single model (SVR, RLR and MLR) at period t.

## Evaluation criteria

The effectiveness of any AI-based model for any type of data-driven algorithm could be evaluated using a number of different metrics by comparing measured and computed data^34,35^ The models are tested during the verification step, which is done utilizing different external and internal validation procedures. Much research uses k-fold cross-validation as one of these approaches^31,36–38^. Similar to this, the computerized model’s substantiation within the scope of its applicability has an appropriate level of precision in line with the application that the model is intended for^39^. As a result, the model’s validation techniques should provide the observed levels of agreement between the projected model and the experimental results as well as the model’s prediction accuracy. In this study, external validation was done using the k-fold cross-validation method before any modeling was done^30^. This approach has also been adopted in other works^40–42^. The coefficient of determinacy (R^2^) and correlation coefficient (CC) were employed as indicators of quality of fit, and two statistical errors, Root mean square error (RMSE) and Mean square error (MSE), were used to evaluate model performance. Equations (10–12) were used to evaluate these parameters.

I. Determinacy coefficient

9 $${{\text{R}}^2 = 1 - \left[ {\frac{{\mathop \sum \nolimits_{{{\text{i}} = 1}}^{{\text{N}}} ({\text{CS}}_{{{\text{com}},{\text{i}}}} { } - {\text{ CS}}_{{{\text{pre}},{\text{i}}}} )^{2} }}{{\mathop \sum \nolimits_{{{\text{i}} = 1}}^{{\text{N}}} ({\text{CS}}_{{{\text{com}},{\text{i}}}} { } - { }\overline{{{\text{CS}}_{{{\text{com}}}} }} )^{2} }}} \right]\;( - \infty \, < R^{2} < \, 1)}$$

II. Correlation coefficient

10 $${\text{CC}} = \frac{{\mathop \sum \nolimits_{{{\text{i}} = 1}}^{{\text{N}}} \left( {{\text{CS}}_{{{\text{com}},{\text{i}}}} { } - { }\overline{{{\text{CS}}_{{{\text{com}}}} }} } \right){ }\left( {{\text{CS}}_{{{\text{pre}},{\text{i}}}} { } - { }\overline{{{\text{CS}}_{{{\text{pre}}}} }} } \right){ }}}{{\sqrt {\mathop \sum \nolimits_{{{\text{i}} = 1}}^{{\text{N}}} ({\text{CS}}_{{{\text{com}},{\text{i}}}} { } - { }\overline{{{\text{CS}}_{{{\text{com}}}} }} )^{2} { }\mathop \sum \nolimits_{{{\text{i}} = 1}}^{{\text{N}}} ({\text{CS}}_{{{\text{pre}},{\text{i}}}} { } - { }\overline{{{\text{CS}}_{{{\text{pre}}}} }} )^{2} { }} { }}}\;\left( { - 1 \, < \, CC \, < \, 1} \right)$$

III. Mean square error

11 $${\text{MSE}} = \frac{1}{{\text{N}}}{ }\mathop \sum \limits_{{{\text{i}} = 1}}^{{\text{N}}} ({\text{CS}}_{{{\text{com}},{\text{i}}}} - {\text{ CS}}_{{{\text{pre}},{\text{i}}}} )^{2} \;(0 \, < \, MSE \, < \infty )$$

IV. Root mean square error

12 $${\text{RMSE}} = \sqrt {\frac{1}{{\text{N}}}{ }\mathop \sum \limits_{{{\text{i}} = 1}}^{{\text{N}}} ({\text{CS}}_{{{\text{com}},{\text{i}}}} - {\text{ CS}}_{{{\text{pre}},{\text{i}}}} )^{2} } \;\left( {0 \, < \, RMSE \, < \, \infty } \right)$$

where ${\text{CS}}$ is the compressive strength, and ${{\text{CS}}}_{{\text{pre}},{\text{i}}}$, ${{\text{CS}}}_{{\text{com}},{\text{i}}}$, $\overline{{{\text{CS}} }_{{\text{pre}}}}$ and $\overline{{{\text{CS}} }_{{\text{com}}}}$ i are the anticipated and calculated values with their respective averages for N data points. Furthermore, the recommended models with the best R^2^, CC, and lowest MSE and RMSE values were nominated for improved prediction within the study domain.

## Results and discussion

### Preliminary analysis

A total of 3323 sets of experimental data variables from published works were collected, processed and used for model training and testing phases to predict the compressive strength of the ternary concrete. Table 1 contains a list of the eight input and one target variables (experimental design variables) that constitutes the database utilized in the ML study. Table 1 summarizes the results of each variable set and the statistical analysis of the datasets (input and output variables) used for model creation.

**Table 1:** Descriptive Statistic of the raw data.

Variables	Mean	SD	Kurtosis	Skewness	Minimum	Maximum
Cement	285.0492	134.6938	1.246711	0.982616	35	778
FA	76.04141	81.54012	−0.2021	0.716373	0	525
GGBFS	89.23807	100.2214	−0.32211	0.837455	0	456.4
Water	178.7962	31.39536	3.192479	1.056575	105	390.39
SP	10.78673	46.85507	83.97302	8.729401	0	602
CA	968.4048	146.4395	1.345375	−0.91398	436	1338.64
F_ag_	755.0354	134.0774	5.106738	−0.73265	191.5	1293
Age	52.67112	78.46571	12.38537	3.319474	1	570
CS	41.64946	21.14666	0.392478	0.704229	0	125

**Figure 4:**
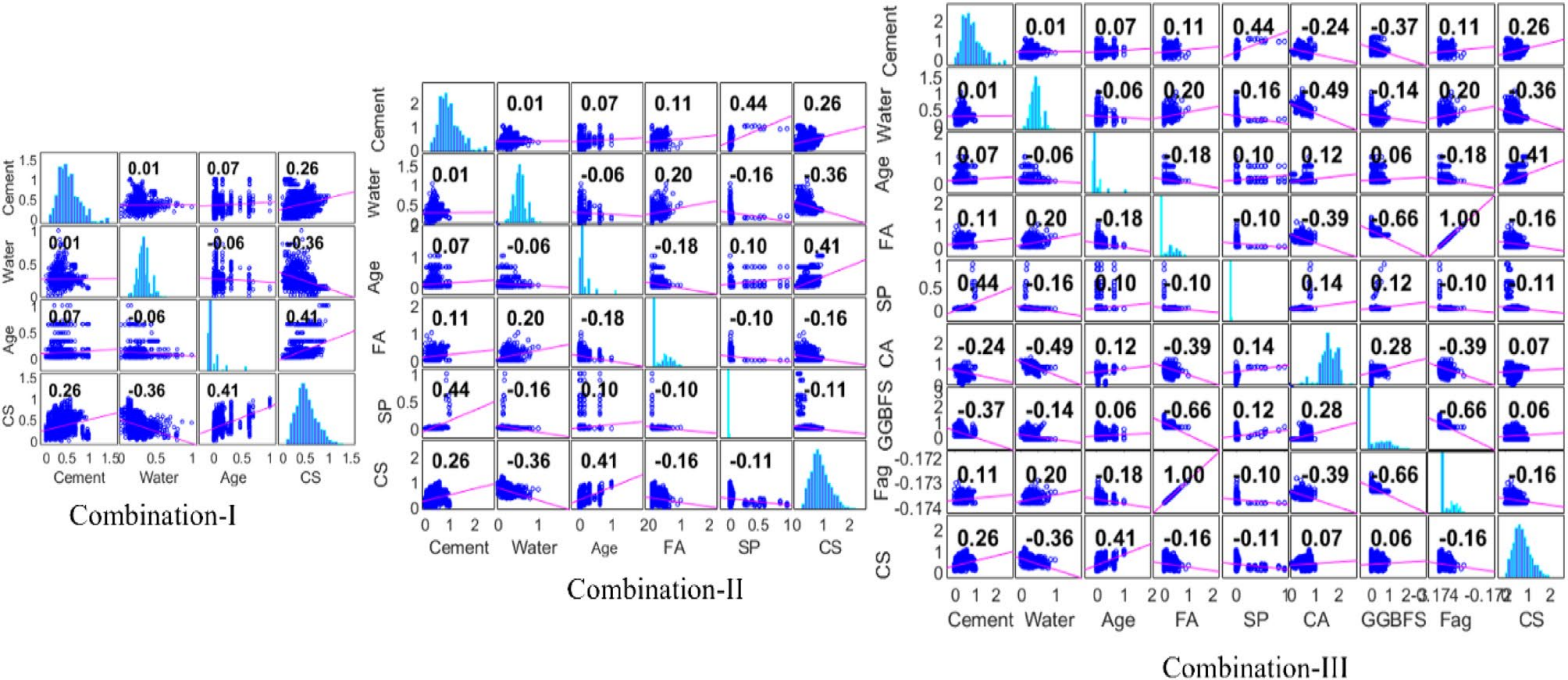
Input variables combination based on correlation analysis.

The Pearson correlation coefficient, which is a ratio of the covariance of two parameters to the product of their standard deviations, was computed to evaluate the simple connection between any two parameters in Table 1. Figure 4 reveals the data distribution and multiple statistical correlation matrix due to interaction between the inputs and output variables, as well as the inter- and intra-dependencies between the input variables. In some ways, the multiple correlation matrix reveals the cross-functional structure of variables based on the common relationship through correlation coefficient values (a number between − 1 and 1): numbers increasing towards either extreme (− 1 and 1) established more correlations between the variables. In order to optimize the input combinations for accurate prediction, different combinations of the input variables were considered, which led to the three different correlation matrices (Fig. 3).

The input combinations were achieved following the deterministic approach so as to optimally influence the input variables to achieve prediction as close as possible to the concrete’s compressive strength. The accuracy of an ML intelligent technique hinges on how well the input variables combines and trains to estimate the compressive strength of the blended concrete. The three combinations used in training the kernel-based models are one (inputs: cement, water and age|output: CS), two (inputs: cement, water, FA, SP and age|output: CS) and three (inputs: cement, water, FA, SP, CA, GGBFS and F_ag_|output: CS). Except for some of the input variables, there are distinct and strong correlations between the selected variables. For example, considering combination-I, the strongest correlations recorded were between age and CS, water and CS, and cement and CS. Though the correlations are low, correlations have largely been established with target variables not much between the input variables. A perfunctory look at the correlation matrix for combination-II revealed the combination-I most correlated variables with CS [R^2^ = 0.26, -0.36 and 0.41 for cement, water and age respectively], and in addition is a relatively better correlation between cement and SP [R^2^ = 0.44]. The correlation strength of the variables recorded with combination III is no different from those recorded in the other combinations (I and II). The majority of the correlation coefficients are too low (no appreciable correlation), while the appreciable correlations are all less than 0.5, which itself is a low correlation.

### Results of AI-based and linear models

Shown in Table 2 are the performance comparison values of the three kernel-based ML models (SVR, RLR and MLR) proposed in this study. Noticeable is the higher R^2^ values of the proposed models during the testing phase when compared with similar values during the training phase albeit with some appreciate difference. During the training phase for all the models, MLR-M2 (Combination-II) had the highest R and R^2^ values though with slight discrepancies from other models’ values. Moreover, the MSE and RMSE values of SVR-M2 (Combination-II) are the lowest. Prior to the decision on the best among the proposed ML models, their predictive performances, which are more important, need to be evaluated. From Table 2, the SVR-M3 (Combination III) had the highest R^2^ values in all the three models with the different combinations. Moreover, the SVR-M3 model’s MSE and RMSE values were the lowest, which are in agreement with those of R^2^ values recorded. As a result, in general, all the kernel-based models (SVR, RLR and MLR) performed well with very good predictions of the target variable (compressive strength). The kernel-based SVR model trained using combination III (SVR-M3) datasets had the most favorable performance with the highest R and R^2^ values and lowest MSE and RMSE values.

**Table 2:** Results of a single ML-based and linear models.

	Training phase				Testing phase			
	R^2^	MSE	RMSE	R	R^2^	MSE	RMSE	R
SVR-M1	0.8253	0.0084	0.0916	0.90845	0.9278	0.0079	0.0889	0.96321
SVR-M2	**0.8388**	**0.0036**	**0.0601**	**0.91586**	0.9753	0.0028	0.0526	0.98757
SVR-M3	0.7326	0.006	0.0774	0.85589	**0.9834**	**0.0019**	**0.0432**	**0.99167**
RLR-M1	0.7703	0.0141	0.1188	0.87769	0.8717	0.0137	0.1171	0.93366
RLR-M2	0.8116	**0.0094**	**0.0969**	0.90089	0.8707	0.0138	0.1175	0.93311
RLR-M3	**0.8503**	0.0111	0.1056	**0.92209**	**0.8776**	**0.0131**	**0.1146**	**0.9368**
MLR-M1	0.8473	0.0118	0.1087	0.92048	0.8614	0.0147	0.1213	0.92813
MLR-M2	**0.8586**	**0.0093**	**0.0963**	**0.9266**	0.8738	0.0135	0.1161	0.93477
MLR-M3	0.8537	0.0104	0.1019	0.92394	**0.8804**	**0.0128**	**0.1133**	**0.93828**

Bolded model evaluation values represent the best input combination out of three for each proposed model.

**Figure 5:**
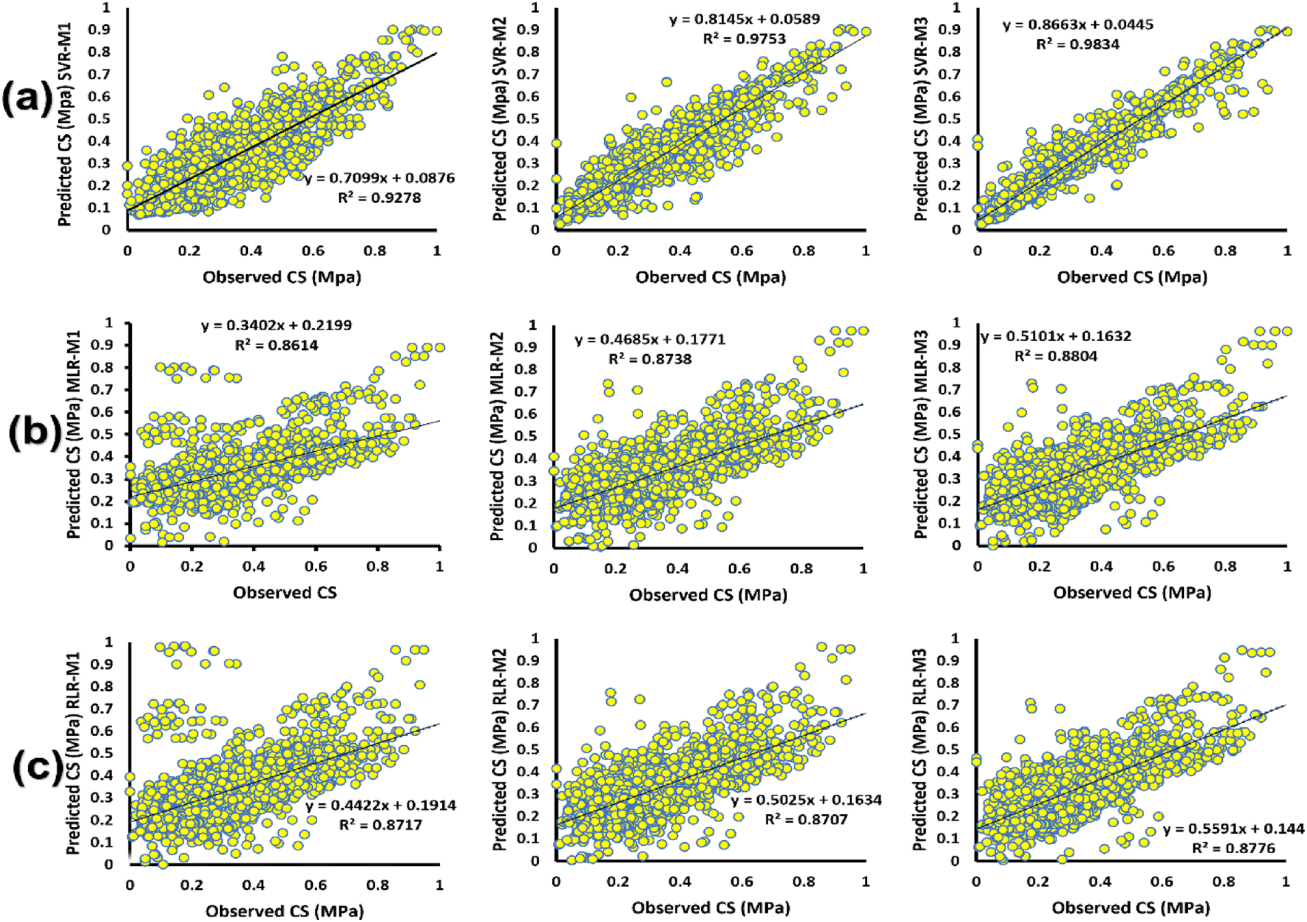
Scatter plot of observed and simulated CS (MPa) (**a**) MLR (**b**) RLR (**c**) SVR.

Depending on the predictive strength of the models, the scatter plots (Fig. 5) are used to test the models using different combinations of input features to reveal variations in predictions of CS from experimental data. In other words, comparative evaluation of the models’ predictive performance was assessed. The goal of the comparative evaluation was to assess the deviation of predicted outcomes from experimental datasets to validate the models used in estimating the concrete’s CS. The analysis revealed that the normalized predicted CS values by the models with the different input combinations accurately tracked and followed the path of the experimental data.

**Figure 6:**
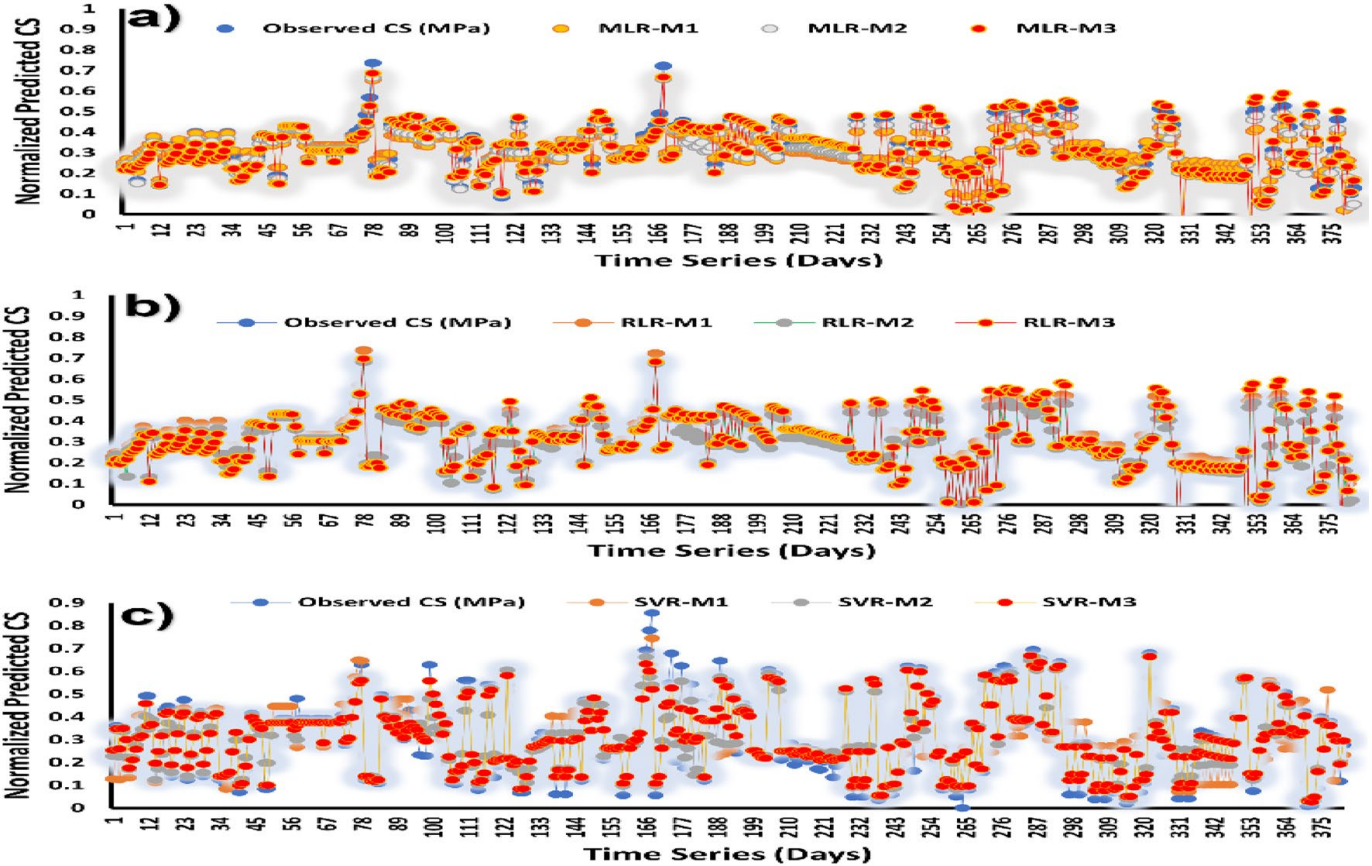
Time series plot between the observed and simulated CS (MPa) (**a**) MLR (**b**) RLR (**c**).

To further reveal the inherent prediction strengths of the proposed models, the predicted output values were plotted against the observed (experimental) results in a time series plot (Fig. 6). The prediction accuracy of the models is evaluated using R^2^, with the highest R^2^ = 0.9834 recorded with SVR-M3 followed by SVR-M2 (R^2^ = 0.9753) and SVR-M1 (R^2^ = 0.9278). The true robustness of SVR is revealed in its excellent ability to generalize, which allows to accurately predict the compressive strength of concrete irrespective of the number of the features combined as inputs.

**Figure 7:**
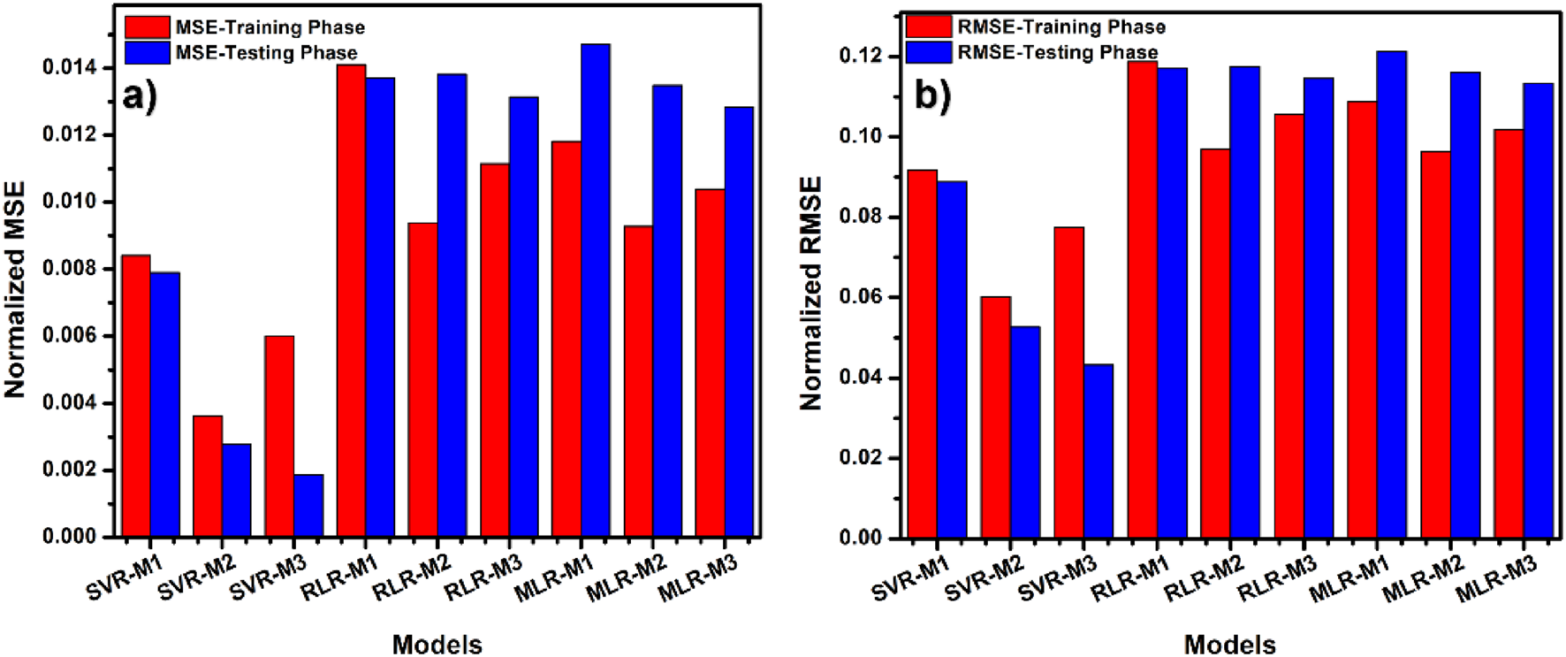
Error plot of observed and simulated CS (Mpa) (**a**) MSE (**b**) RMSE.

In addition to other plots, the prediction error plots (Fig. 7) are also essential to allow the data scientists to gauge the models’ level of variance from the expected experiment values. A general look at Fig. 6 reveals that the least errors were recorded with the SVR models in all combinations during the testing and training phases. This one again proves the inherent computational power of SVR, which is good in acknowledging the presence of non-linearity in the data thus providing the prediction model proficiency. It can do this because of its ability conveniently find a hyperplane in an n-dimensional space to classify the datasets. SVR-M3 has the least normalized MSE and RMSE values from the error plot, which goes to proves that it has the best predictive ability among the proposed models. It is also worthy to note that the third combination (M3) in all the models has the least deviation or variance (errors) from the experimentally observed compressive strength values of the blended concrete.

### Result of nonlinear simple averaging

**Figure 8:**
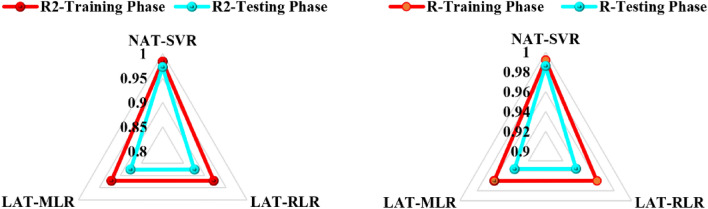
Radar plot for NAT and LAT based on R^2^ and R.

This section was motivated by several theories, one of them is the no-free lunch theorem which is based on the fact that models behave differently owing to the different response to input–output variables. For this reason, it is paramount to have a common ground in any modelling schema that would couple the strengths and weaknesses of the predictive results and enhance the predictive accuracy. Table 3 presented the prediction results for the three averaging techniques (NAT-SVR, LAT-RLR, and LAT-MLR). The outcomes show that nonlinear averaging outperforms the two linear averaging approach based on the performance criteria. This is not surprising as nonlinear methods were reported to be superior to the traditional linear models. Another merit of nonlinear methods over traditional models is the tuning of hyperparameters, the inability to extrapolate and control several of its parameters has been iterated in previous literature. The advantage of internal and external validation made nonlinear AI-based models satisfactory to capturing the complex responses, unlike classical models that consistently fail to present the real scenario due to validation problems. The numerical assessment of the predictive models proved that NAT-SVR, LAT-RLR, and LART-MLR attained reliable accuracy of 97.2%, 87.5%, and 87.6%, respectively in the verification phase. The goodness-of-fit is presented in Fig. 8 as the radar plot.

**Table 3:** Results of nonlinear- and linear averaging methods.

	Training phase				Testing phase			
	R^2^	MSE	RMSE	R	R^2^	MSE	RMSE	R
NAT-SVR	0.9839	0.0006	0.0235	0.9919	0.9722	0.0013	0.0357	0.98601
LAT-RLR	0.9211	0.0005	0.0227	0.95972	0.8754	0.0011	0.0339	0.93565
LAT-MLR	0.9214	0.0005	0.0227	0.9599	0.8761	0.0011	0.0339	0.93603

**Figure 9:**
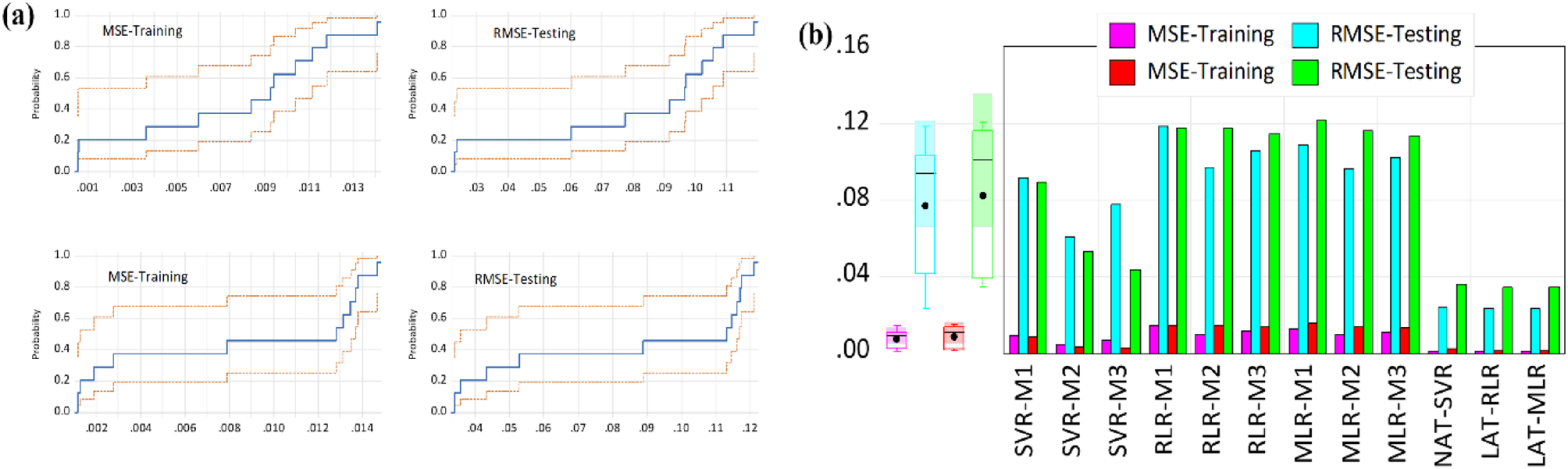
Comparison of single, NAT, and LAT models (**a**) cumulative probability (**b**) error plot.

Similarly, the error justification indicated that RMSE of NAT-SVR is the lowest with the value of 0.357 follow by the two linear averaging with the barrack value of 0.0339. The degree of freedom in nonlinear AI-based models permits them to inculcate the generalization ability unlike traditional and physics-based models associated with overparameterization^43–45^. In this context, AI-based models were set as a benchmark approach to address several civil engineering problems against the conceptual and traditional learning-based methods. The soft computing-oriented modelers were motivated by the ability of soft techniques and scientific hypotheses to produce reliable hydrological estimations such as stream flow. To understand the pattern of the predictive outcomes’ cumulative probability and details error visualization are presented in Fig. 9a,b. According to^46–49^ standalone model can outperform hybrid or ensemble models in rare cases but the generalization and sharing ability of the strength and weakness together late is better than the former.

## Conclusions

The study deployed kernel-based models (SVR, RLR and MLR) bearing in mind the additional nonlinear dimension offered the concrete by introducing fly ash and ground granulated blast furnace slag to cement, to predict the compressive strength of the developed ternary-based concrete. In the study, nine proposed models were developed with three different input features combinations for each model and their prediction performances were comparatively evaluated in predicting the compressive strength of concrete. The mixture compositions as inputs (FA, GGBFS, OPC, SP, water, age, CA and F_ag_) supplied for training were employed to estimate the compressive strength of the blended concrete. Using statistical apparatus (R^2^, R, MSE and RMSE), the suggested model testing performance in strength estimation were evaluated in deciding in the best performing model and with which feature combination. The SVR-M3 (SVR-combination III) model, with the lowest errors (MSE = 0.0019 and RMSE = 0.0432) and highest R^2^ = 0.98345 and R = 0.99167 values, which in terms of estimating the compressive strength outperformed the other two suggested models.

This viewpoint is supported by the predicted performance of models’ outcomes covered in “Results and discussion” section. The controlling combination input variables are cement, water, fly ash, superplasticizer, coarse aggregate, fine aggregate, and ground granulated blast furnace slag. Notably, removing non-contributing input features and including mainly controlling input features in training the SVR algorithms enhanced the model’s prediction accuracy and decreased modeling errors in all three kernel-based models. These models outperformed previous ML models used to estimate the compressive strength of ternary-blended concrete. According to our findings, kernel-based regression models and nonlinear ensemble models are potential new techniques for forecasting concrete compressive strength. These models may be used to enhance the accuracy of concrete mix designs and assure the safety and reliability of concrete buildings. Furthermore, given the environmentally motivated drive to use waste materials in concrete mixtures, it would be reasonable to regularly update the model to make it compatible for newly developed composite materials compositions.

## Data Availability

The datasets used and/or analysed during the current study available from the corresponding author on reasonable request.
